# [Corrigendum] Establishment of hypoxia induction in an *in vivo* animal replacement model for experimental evaluation of pancreatic cancer

**DOI:** 10.3892/or.2023.8626

**Published:** 2023-09-11

**Authors:** Nathalie Bauer, Li Liu, Ewa Aleksandrowicz, Ingrid Herr

Oncol Rep 32: 153–158, 2014; DOI: 10.3892/or.2014.3196

Subsequently to the publication of the above paper, an interested reader drew to the authors' attention that, on p. 156, the data panels shown to represent the ‘CoCl_2_’ and ‘TRIP’ data panels in Fig. 3 for the DAPI experiments were apparently the same, even though different experiments were being depicted here.

The authors were able to re-examine their original data files, and realized that this figure had been assembled incorrectly: there was an inadvertent mix-up of a pair of the DAPI control images. The revised version of Fig. 3, containing the correct DAPI data for the ‘TRIP’ experiment, is shown opposite. Note that the revisions made to this figure do not affect the overall conclusions reported in the paper. The authors are grateful to the Editor of *Oncology Reports* for allowing them the opportunity to publish this Corrigendum, and apologize to the readership for any inconvenience caused.

## Figures and Tables

**Figure 3.**
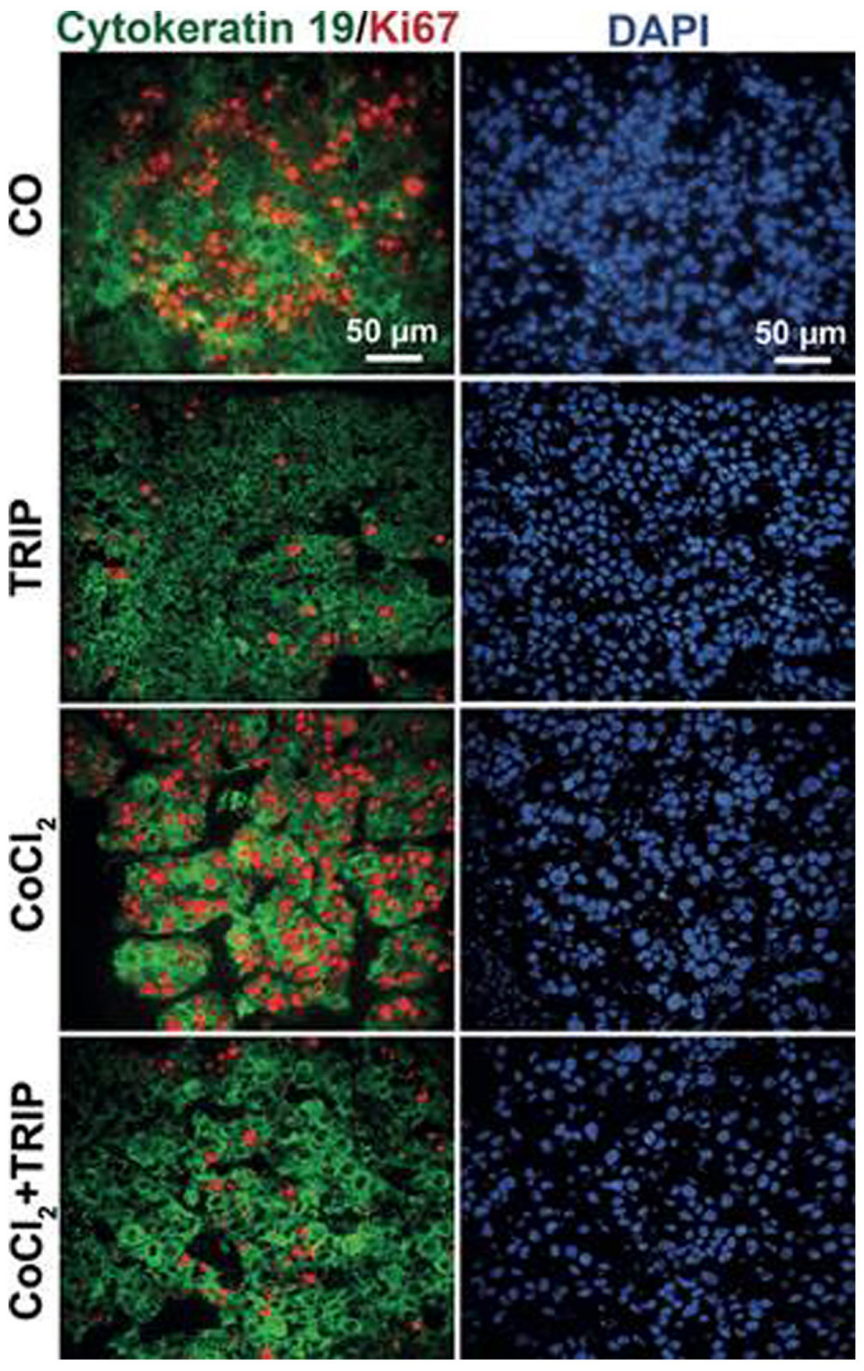
The human markers cytokeratin 19 and Ki67 ensure induction of hypoxia in human xenograft cells. Representative images of double immunofluorescence stainings of frozen tumor xenograft sections (×400, magnification) with the proliferation marker Ki67 (red) and the cytoskeletal marker cytokeratin 19 (Cyt19, green); both detect specifically human cells. Nuclei were stained with DAPI (blue). The bar indicates 50 µm.

